# A novel virus that infecting hypovirulent strain XG36-1 of plant fungal pathogen *Sclerotinia sclerotiorum*

**DOI:** 10.1186/1743-422X-6-96

**Published:** 2009-07-07

**Authors:** Liyan Zhang, Yanping Fu, Jiatao Xie, Daohong Jiang, Guoqing Li, Xianhong Yi

**Affiliations:** 1 State Key Laboratory of Agricultural Microbiology, Huazhong Agricultural University, Wuhan 430070, Hubei Province, PR China; 2 The Provincial Key Lab of Plant Pathology of Hubei Province, College of Plant Science and Technology, Huazhong Agricultural University, Wuhan, 430070, Hubei Province, PR China

## Abstract

**Background:**

*Sclerotinia sclerotiorum *is a notorious plant fungal pathogen which spreads across the world. Hypovirulence is a phenomenon where the virulence of fungal pathogens is decreased, even lost, due to mycovirus infection. The potential of hypoviruses for biological control of the chestnut blight fungus (*Cryphonectria parasitica*) has attracted much interest, and has led to discovery of new hypovirulent strains in other fungi.

**Results:**

A hypovirulent strain, strain XG36-1, was isolated from a typical lesion on the stem of rapeseed (*Brassica napus*) caused by *Sclerotinia sclerotiorum*. Strain XG36-1 grew on PDA very slowly (average 2.5 ± 0.1 mm/d) with sectoring, and developed abnormal colony morphology with few sclerotia. Unlike health strains (such as wildtype strain XG-13), it was unable to induce lesions on detached leaves of rapeseed. Sclerotia of strain XG36-1 produced apothecia rarely. A sexual progeny test showed that the phenotypes of all 104 sexual progeny were not different from wildtype strain XG-13 which shows normal phenotype of *S. sclerotiorum*, and protoplast regeneration tests showed that 25.5% of the regenerants of strain XG36-1 were recovered fully. Furthermore, the hypovirulence and its associated traits could be transmitted to XG36-1A34^*R*^, a hygromycin-resistance gene labelled sexual progeny of strain XG36-1, by hyphal anastomosis. Transmission electron microscope (TEM) observation showed that the cytoplasm of strain XG36-1 was destroyed and granulated; the membranes of nuclei and mitochondria were disintegrated; and mitochondrial cristae were cavitated. Viral particles (about 40 nm) in hyphae of strain XG36-1, but not in its sexual progeny and wildtype strain XG-13, could be observed with TEM, and several virus-like particles were uniquely enveloped by single layer membrane in the cells of strain XG36-1. Furthermore, the viral particles could be co-transmitted with the hypovirulence traits through hyphal anastomosis.

**Conclusion:**

Hypovirulence and its associated traits of strain XG36-1 could be mediated by a fungal virus. Currently, we could not know the characteristic of this virus, but it likely represent a new type of mycovirus in *S. sclerotiorum*, and possibly in fungi.

## Background

Rapeseed (*Brassica napus*) is one of the most important oilseed crops, and offers the potential for biodiesel production to relieve the pressure of the current energy shortage. The area planted with rapeseed in China is currently 7.4 million hectares, and the Chinese government encourages farmers to plant more winter rapeseed during late fall to early summer in central China . *Sclerotinia sclerotiorum *(Lib.) de Bary is an important fungal plant pathogen which damages a wide variety of crops throughout the world [1]. In China, this fungus causes stem rot of rapeseed and is responsible for serious losses every year; in 2008, more than 15–70% of rapeseed plants were killed by this pathogen in Hubei Province. Due to the shortage of disease-resistant cultivars, chemical control is currently the only choice to control stem rot. However, there are problems associated with chemical control of stem rot. Firstly, fungicide control requires treatment during the bloom stage of rapeseed, but this is not practical because the chemical does not arrive at the stems efficiently through heavy canopy. Secondly, fungicide-resistant strains of *S. sclerotiorum *have been frequently isolated in the field [2]. Non-fungicidal alternatives for the control of stem rot of rapeseed are necessary.

Hypovirulence or hypovirulence is a phenomenon where the virulence of fungal pathogens is decreased, even lost, due to mycovirus infection. Hypovirulence was first reported in the chestnut blight, a destructive disease caused by *Cryphonectria parasitica *by Grente [3]. The successful control of chestnut blight with hypovirulent strains of *C. parasitica *represented an alternative approach to biological control fungal diseases other than with mycoparasites and antagonists [4,5]. The potential of hypovirulence for biological control of plant fungal diseases has attracted much interest, and has lead to discovery of new hypovirulent strains in other fungi. Other mycoviruses causing hypovirulence or hypovirulence of plant fungal pathogens include: mitovirus in *C. parasitica *[6], *Ophiostoma novo-ulmi *[7], *Sclerotinia homoeocarpa *[8,9], *Chalara elegans *[10] and *Botrytis cinerea *[11]; mycoreoviruses in *C. parasitica *[12], and *Rosellinia necatrix *[13]; and some unclassified mycoviruses, such as SsDRV in the family *Flexiviridae *in *S. sclerotiorum *[14], DaRV in *Diarporthe ambigua *[15], FgV-DK21 in *Fusarium graminearum *[16,17] and a 33-nm isometric mycovirus in *B. cinerea *[18].

Hypovirulent strains have been reported in *S. sclerotiorum*, such as isolate 91, strain Ep-1PN and isolate S10 [19-21]; and mycoviruses that associated with hypovirulence of *S. sclerotiorum *were isolated from strain Ep-1PN [16,22]. Hypovirulence in *S. sclerotiorum *is likely common since we often isolate some mild strains, even non-virulence strains, from fields. In this paper, we report on a hypovirulent strain isolated from a typical lesion on stem of rapeseed and called XG36-1 which possibly differs from previously reported hypovirulent strains of *S. sclerotiorum*.

## Results

### Strain XG36-1 showed hypovirulence phenotype

**Figure 1:**
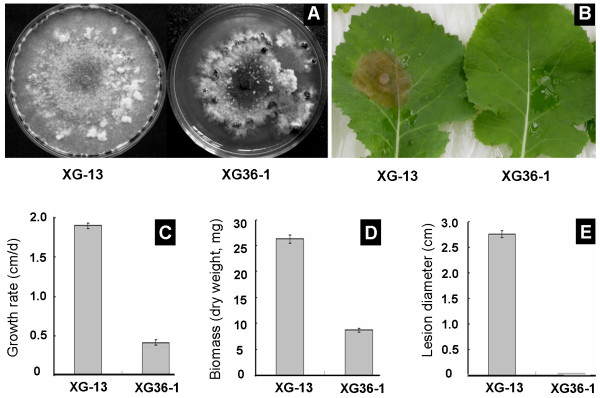
**Hypovirulence and its associated traits of *Sclerotinia sclerotiorum *strain XG36-1**. A, abnormal colony morphology developed on 20 ml PDA plate at 20–22 C for 15 days, a typical colony morphology of *S. sclerotiorum *(strain XG-13) developed at the same condition was showed as control. B and E, strain XG36-1 did not induce any typical lesion on detached leaf of rapeseed (*Brassica napus*) as strain XG-13 did; lesions were photographed and measured at 60 hpi. C and D, comparing to strain XG-13, strain XG36-1 grew on PDA plate slowly and produced less biomass (grown out on cellophane membranes on top of 20 ml PDA plate at 20°C for 72 h).

The colony of strain XG36-1 on PDA was thick with many sectors at the colony margin; only a few sclerotia were produced and distributed in the colony irregularly. The colony morphology of strain XG36-1 was abnormal and obviously different from strain XG-13, a healthy wildtype strain isolated from the same filed as strain XG36-1 (Fig 1A). Hypha extended slowly on PDA plate, and the hyphal tips often branch excessively. The growth rate of XG36-1 was 4.1 ± 0.1 mm/d, which was significantly lower than 19.0 ± 1.1 mm/d found for a wildtype strain XG-13 (Fig 1C). The biomass produced by strain XG36-1 on a 9-cm-diam. PDA plate was 8.6 ± 0.1 mg after 72 h incubation, while that produced by strain XG-13 was 26.3 ± 2.1 mg (Fig 1D). Unlike strain XG-13, strain XG36-1 produced fewer sclerotia in the mature colony, with the average number of sclerotia at 5 sclerotia/plate, while strain XG-13 had 12 sclerotia/plate. Strain XG36-1 was almost incapable of inducing any lesions on detached leaves of rapeseed at 60 h post inoculation (hpi), while wildtype strain XG-13 could induce typical lesions on detached leaves, averaging 2.75 ± 0.14 cm at 60 hpi (Fig 1B, E). Thus, the strain XG36-1 was judged to be a hypovirulent strain of *S. sclerotiorum*.

### Multi-phenotype of protoplast regenerants of strain XG36-1

**Figure 2:**
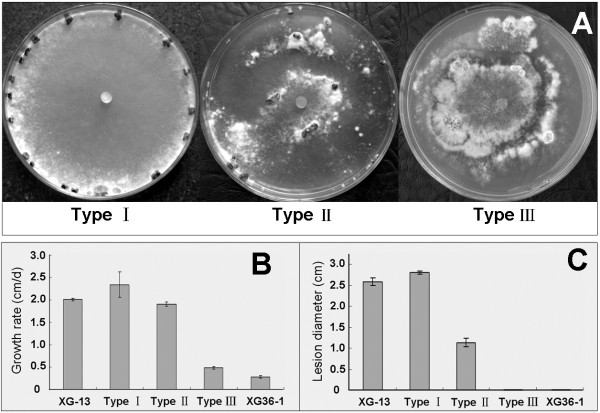
**Diverse phenotypes of protoplast regenarants of *S. sclerotiorum *hypovirulent strain XG36-1**. A, three types of colony morphologies, namely Type I, Type IIand Type III, were developed on PDA plate at 20–22 C for 15 days. Regenarants in Type Iwere not significantly different from wildtype strain XG-13, while regenarants in Type III were similar to strain XG36-1 (see Figure 1), Type II were intermediate type between Type Iand Type III. B and C, comparison of the hyphal growth rate on PDA plate and virulence on detached rapeseed leaves among three types of regenarants. The growth and virulence of regenarants in TypeI were fully recovered, and that in Type II were partially recovered; the growth and virulence of regenarants in Type III was not significantly different from strain XG36-1.

Fifty-five protoplast regenerants of strain XG36-1 were obtained. Their growth rates, colony morphology and pathogenicity were tested, and the results showed that the phenotypes of these regenerants were significantly diverse. Based on growth rate and colony morphology, these regenerants could divide into three groups, namely TypeI, TypeII and Type III. Type I regenerants grew on PDA just like wildtype strain XG-13, developing normal colony morphology of *S. sclerotiorum*, and capable of inducing typical lesions on detached leaves of rapeseed (Fig 2). Approximately 25.5% of the regenerants (14/55) belonged to Type I. Type II regenerants grew on PDA much faster than hypovirulent parental strain XG36-1, but slower than wildtype strain XG-13. These regenerants could cover an entire 9-cm-diam. PDA plate by 14 days with an average growth rate of 9.7 mm/d. These regenerants could induce small lesions on detached leaves with an average size of 1.0 cm across (Fig 2). Approximately 23.8% of the regenerants (13/55) belonged to type II. Type III regenerants were not significantly different from hypovirulent parental strain XG36-1 (Fig 2), comprising 51.7% of the regenerants (28/55).

### Normal phenotype of sexual progeny of strain XG36-1

**Figure 3:**
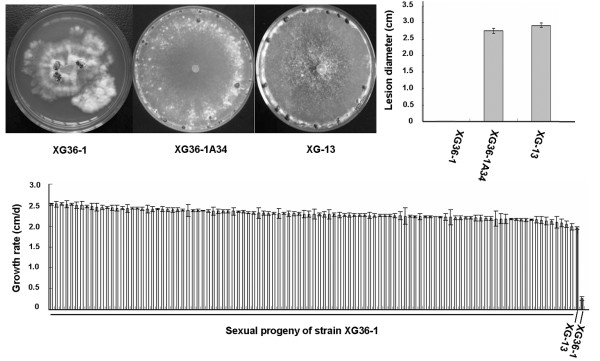
**Sexual progeny of hypovirulent strain XG36-1 showed normal phenotypes of *S. sclerotiorum***. A and B, the colony morphology and virulence on rapeseed detached leaves of a randomly selected sexual progeny XG36-1A34; C, the growth rate of 104 tested sexual progeny.

Only a few of sclerotia of strain XG36-1 could be successfully induced to form apothecia. 104 single-ascospore-isolation sexual progeny were obtained. The cultural characteristics and pathogenicity of these 104 sexual progeny were tested, and the results showed that all sexual progeny had a typical wildtype phenotype of *S. sclerotiorum*. Compared to strain XG-13, the grow rate, colony morphology of sexual progeny were not significantly different (Fig 3).

### Transmission of hypovirulence phenotype of strain XG36-1

**Figure 4:**
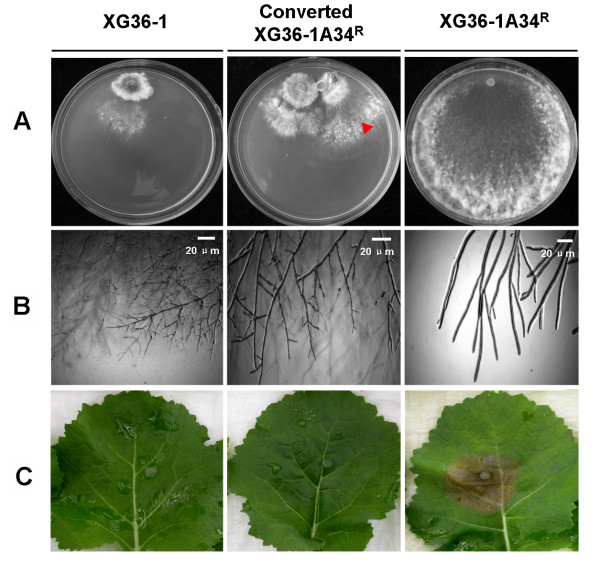
**Transmission of hypovirulence and its associated traits of strain XG36-1**. A, the colony of hygromycin-resistant gene labelled strain XG36-1A34^R ^was converted when dual culturing with hypovirulent strain XG36-1(red triangle). B, the hyphae at the colony margin of strain XG36-1A34^R ^branched excessively as strain XG36-1 did. C, converted strain XG36-1A34^R ^also lost virulence on detached rapeseed leaves.

After contacting with strain XG36-1 on PDA, the hyphae around the colony margin of hygromycin-resistance gene labelled sexual progeny XG36-1A34^R ^branched excessively (Fig. 4), subcultures from this region showed hypovirulence traits (Fig 4). The growth rate, the sectoring, the colony morphology, and the pathogenicity of infected XG36-1A34^R ^were not significantly different from strain XG36-1. Furthermore, the hypovirulence phenotype obtained by XG36-1A34^R ^could be transmitted to XG36-1A34 and other sexual progeny.

### Virus particles observed in strain XG36-1

**Figure 5:**
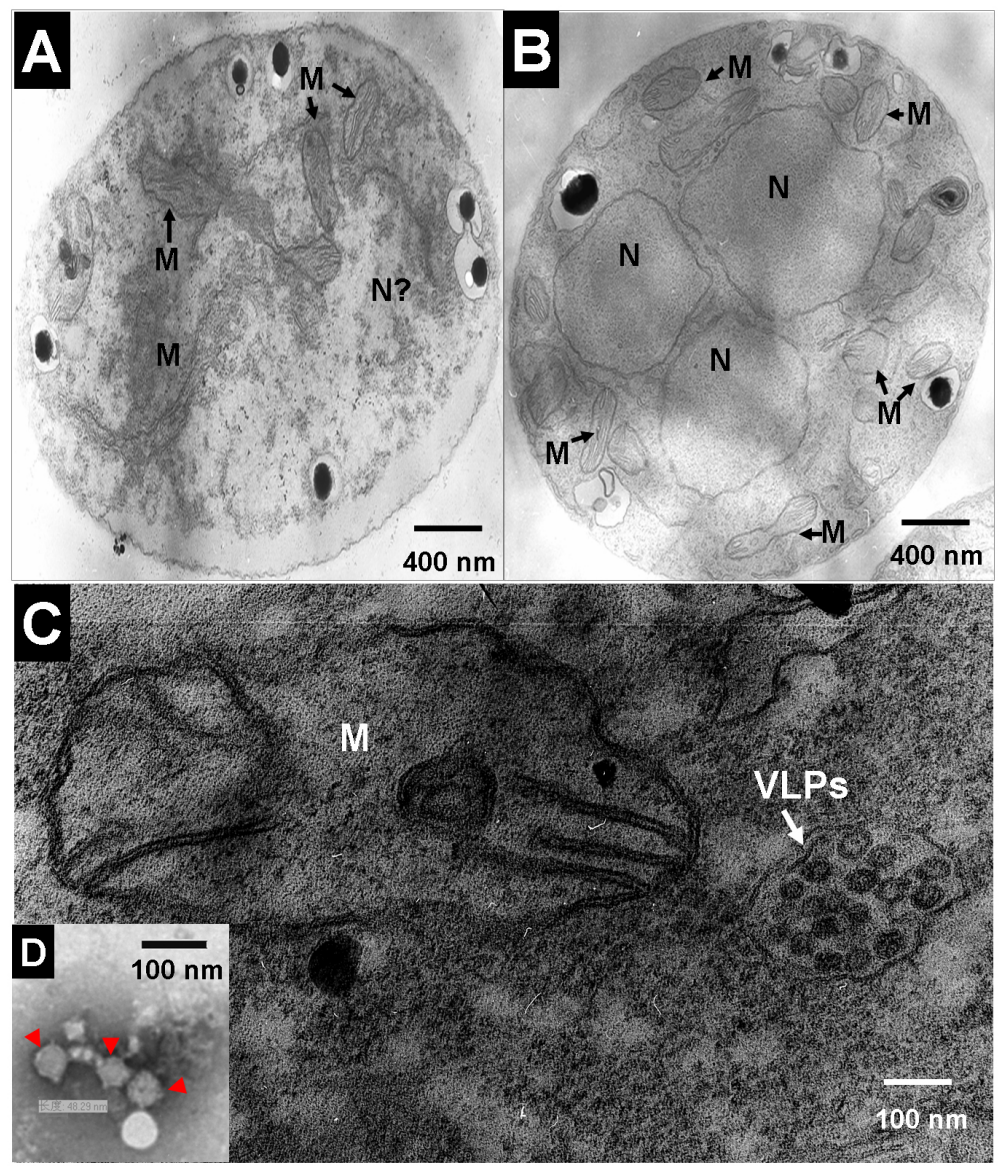
**Ultrastructure and virus-like particles (viral particles) in the cell of *S. sclerotiorum *hypovirulent strain XG36-1 observed under transmission electron microscopes (TEM)**. A, hyphal ultrastructure of hypovirulent strain XG36-1, the cytoplasm was granulated, the membranes of nuclei (N) and mitochondria (M) was disintegrated; only a few mitochondria existed, but the mitochondrial cristae was cavitated. B, hyphal ultrastructure of wildtype strain XG-13, the cytoplasm was well-distributed, plentiful mitochondria, and the membranes of nuclei (N) and mitochondria (M) was not destroyed. C, viral particles (white arrow) in cell of hypovirulent strain XG36-1, the size of individual particle is about 40 nm, several particles were enveloped by single-layer membrane. D, A few viral particles (red triangles) could be observed after negatively stained with 1% uranyl acetate on carbon-coated 400 mesh copper grids. Ultrastructure observation was carried out under FEI Tecnai G^2 ^20 TWIN transmission electron microscope).

Under TEM, the cytoplasm of strain XG36-1 was seen to be destroyed and granulated; the membranes of nuclei and mitochondria were disintegrated. Only a few mitochondria were seen in cell, while, the mitochondrial cristae were cavitated (Fig 5A). However, the nuclei and mitochondria in wildtype strain XG-13 were not destroyed, and the cytoplasm were well-distributed and filled with plentiful mitochondria, nuclei and mitochondria was not destroyed (Fig 5B). Viral particles could be observed in the cells of strain XG36-1, but not in the cells of wildtype strain XG-13 or sexual progeny XG36-1A34. The viral particles were almost isometric, with a diameter of ~40 nm. Several viral particles were enveloped by a single layer membrane (Fig 5C). However, viral particles could be observed in the cells of subcultures of strain XG36-1A34^R ^after contacting the colony of strain XG36-1. Thus, the viral particles are transmissible and associated with hypovirulence of strain XG36-1. Viral particles could also be extracted from the hyphae of strain XG36-1 after ultra-centrifugation in a Cesium chloride (CeCl) gradient medium, but only very few viral particles could be observed through TEM.

### Viral nucleic tides not extracted from strain XG36-1

All attempts to extract dsRNA were not successful. Double-stranded RNA could not be extracted directly from hyphae of strain XG36-1, but could be extracted from previously reported hypovirulent strain Ep-1PN (positive control). No viral RNA sample could be extracted from the pellets precipitated with ultracentrifugation.

## Discussion

Our experiments showed that strain XG36-1 was a hypovirulent strain of *S. sclerotiorum*. The protoplast regenerants test suggested that the hypovirulence-associated element (HAE) in cells of strain XG36-1 did not distribute equally, and hypovirulence of regenerants derived from protoplasts that without DAE or with a low concentration of HAE were cured or partially cured. All tested sexual progeny showed the wildtype phenotype of *S. sclerotiorum *suggesting that chromosomal or DNA changes (nuclear genomic mutations) were not responsible for the hypovirulence of strain XG36-1, and that there is likely some mechanism to eliminate HAE during sexual reproduction. Transmission tests showed that the hypovirulence of strain XG36-1 could be transmitted to wildtype strains efficiently. The hypovirulence and its associated traits of strain XG36-1 is similar to previously reported hypovirulent strain Ep-1PN [14,20,23]. Thus, the HAE in strain XG36-1 could be transmissible genetic elements.

In fungi, both fungal plasmids and mycoviruses are transmissible genetic elements, and both mycoviruses, and some fungal plasmids may cause hypovirulence to their hosts [24]. Fungal plasmids are not likely to be HAE of strain XG36-1. Extra-chromosomal DNA segments were not observed with agarose gel electrophoresis analysis of whole DNA samples (data not shown). Meanwhile, viral particles were observed in hyphae of strain XG36-1, but not in its sexual progeny nor in wildtype strain XG-13, and viral particles were always associated the transmission of hypovirulence traits of strain GX36-1. Furthermore, the observed elimination of viruses and hypovirulence during sexual reproduction of strain XG36-1 is in accord with the sexual reproductive behaviour of virus-infected ascomycetous fungal hosts, where the viruses are not transmitted to progeny [25]. Thus, the transmissible DAE in strain XG36-1 is likely to be a mycovirus.

The viral particles in strain XG36-1 is possibly a new type of mycovirus that infecting *S. sclerotiorum*. The distinct characteristic of the viral particles in strain XG36-1 is that the isometric viral particles are enveloped with a single layer membrane which is possibly derived from its host. Viral particles (double membranes bodies, DMB) have been observed in hypovirulent isolate 275 of *S. sclerotiorum *[26], but not in hypovirulent strain Ep-1PN and isolate S10 [14,27]. Furthermore, the average size of DMBs in isolate 275 was about 70 nm in diameter which was much larger that that of viral particles in strain XG36-1.

This unique envelopment of particles has not been observed in other mycoviruses which may or may not encode coat protein. Viruses infect in all the major groups of fungi kingdom, and RNA viruses in the family *Chrysoviridae*, *Hypoviridae*, *Narnaviridae *(*Mitovirus*), *Partitiviridae *and *Totiviridae *are typical fungal viruses [28]. However, more and more fungal viruses were characterized on molecular level, the plenty diversity of fungal viruses in nature is becoming more and more clear. Viral particles in strain XG36-1 are enveloped uniquely, and could cause severe debilitation of host with low titre, suggest that virus infecting strain XG36-1 is most likely to be a novel mycovirus associated with hypovirulence of plant fungal pathogen.

## Conclusion

Our work, here, proved that the hypovirulence and it associated traits of *S. sclerotiorum *strain XG36-1 could not be transfer to sexual progeny vertically, and the hypovirulence associated element (HAE) is not distributed equally in cells of strain XG36-1. Thus, the hypovirulence of strain XG36-1 is not due to the genome mutation. Hypovirulence and its associated traits could be transferred efficiently to vegetative compatible strain XG36-1A34^R^, a hygromycin resistance gene labelled sexual progeny of strain XG36-1, through hyphal anastomosis. Thus, the HAE in strain XG36-1 is a mobile element.

The cytoplasm of strain XG36-1 was granulated and not well-distributed, the membranes of nuclei and mitochondria were disintegrated; and mitochondrial cristae were cavitated. Viral particles could be observed in cells of strain XG36-1, but not in wildtype strain XG-13 and sexual progeny XG36-1A34. Viral particles could also be extracted with ultracentrifugation from the hyphae of strain XG36-1. Although the viral nucleic acids were not extracted and identified currently, however, comparing to previously reported hypovirulence or debilitation associated mycoviruses, the virus in strain XG36-1 is unique; it is most likely to be a novel mycovirus associated with hypovirulence of plant fungal pathogen.

## Methods

### Fungal strains, media and culture

*S. sclerotiorum *strain XG36-1 was isolated from a typical lesion on stem of rapeseed at Xiaogan County, Hubei Province, P R China. Strain XG-13, a healthy wildtype strain, was also isolated from another typical lesion in the same rapeseed field as strain XG36-1. Hypovirulent strain Ep-1PN was originally isolated from diseased eggplant [21]. All strains and their derivatives were grown on PDA (potato dextrose agar, PDA) at 20°C, and stored on PDA slants at 4–6°C.

### Comparison of cultural characteristics

Strains XG36-1 and XG-13 were maintained on Petri dishes containing 20 ml PDA, and incubated at 20°C for 3 days. To assess growth rates, 5-mm-diameter agar disks from actively growing colony margins of XG36-1 and XG-13 were transferred onto 9-cm-diam Petri dishes containing 20 ml PDA, and then incubated at 20°C. The diameter of colonies of XG-13 and XG36-1 was measured at 24 hour post inoculation (hpi) and 48 hpi, respectively; the hyphal growth rate of the two strains was calculated as follows: growth rate (cm/d) = (48 hpi diam. - 24 hpi diam.)/2. To compare the biomass between XG36-1 and XG-13 produced on PDA, these two strains were grown out on cellophane membranes on top of PDA (20 ml) at 20°C for 48 h, and then the mycelial mass was rolled from the membrane, placed in an 80°C oven for 10 h, and the dry weights were recorded. To compare the colony morphology, the colonies were grown on 20 ml PDA plates at 20°C for up to 15 days.

### Pathogenicity test of XG36-1

Agar disks (5-mm-diam.) from actively-growing colony margins of strain XG36-1 and its derivatives and strain XG-13 were placed on the leaves of rapeseed with the mycelial side facing the leaf surface, and then the inoculated leaves were placed in an incubator at 20°C and 100% relative humility for 60 h. Lesion diameter on each inoculated leaf was measured. There were five replicates for each treatment.

### Protoplast preparation and regeneration

To obtain protoplasts of strain XG36-1, mycelial-agar discs (5-mm-diam.) cut from actively growing colony margins of strain XG36-1 were transferred onto cellophane membranes overlaying PDA. After 2 days, the mycelia were collected from cellophane membranes, and then ground with sterilized mortar and pestle to make hyphal fragments. Approximately 1 ml of hyphal fragment mush was transferred into a 250 ml flask containing 80 ml PDB (Potato Dextrose Broth, PDB), and shaken at 150 rpm for up to 20 h at 20°C. The filtrate was collected by passing through two layers of sterilized cheesecloth, and the mycelium was washed twice with 100 ml of potassium chloride (KCl) buffer (0.6 mol/L). The mycelial mass was squeezed to remove liquids, and then re-suspended with digestion buffer which contained 1.5 mg/ml Lysing enzymes from *Trichoderma harzianum *(Sigma-Aldrich, Inc), and then incubated at 32°C for 3 h. The liquid was filtered through four layers for sterilized cheesecloth and than passed through two layers of sterilized filter paper to remove the debris and undigested hyphal fragments. Protoplasts were collected by centrifugation for 10 min at 4000 rpm. The precipitate was washed twice with 0.6 mol/L KCl solution by re-suspension and centrifugation at 4000 rpm for 5 min. The final precipitate was re-suspended with in 0.6 mol/L KCl solution to give 1–2 × 10^3 ^protoplasts/ml for regeneration. One hundred microliter of protoplast suspension was gently mixed with 20 ml regeneration medium (RM: sucrose 0.7 M; yeast extract 0.5 g/L, agar 1.5 g/L, adding KCl to a final concentration 0.6 mol/L before use), and poured into a Petri dish (diam. 90 mm). The plates were incubated at 20–22°C for 3–4 days, and then small colonies were observed on the RM plates and transferred onto fresh PDA plates. These subcultures were considered as protoplast regenerants of strain XG36-1. The cultural characteristics and pathogenicity of regenerants were tested with measures described above.

### Sexual reproduction and progeny isolation

To collect sclerotia, strain XG36-1 was allowed to grow on sterilized carrot at 20°C for up to one month. After that, sclerotia were harvested and washed with tap water to remove mycelia and debris, and dried at room temperature for up to two weeks. To induce carpogenic germination of sclerotia, the dry sclerotia were placed at -20°C for up to one month, and then the low-temperature treated sclerotia were surface sterilized with 70% ethanol and sowed onto sterilized wet sand and incubated at 15–17°C for up to two months. A few sclerotia then produced apothecia. To obtain ascospores, mature apothecia were placed into a 50 ml syringe with 10 mL sterile water, and then the syringe was capped with silica gel, and then the piston was pushed and pulled several times to allow apothecia to release ascospores. The ascospores suspension was collected and adjusted to a concentration of ~10^3 ^spores/ml. To create mono-ascospore cultures, 200 μl of the spore suspension was spreaded over a thin layer of water agar (10 ml water agar in a 90-mm-diam. plate), and then placed at 20°C for 24 h Under light microscopy, the mycelium formed from a single ascospore was excised, and transferred to fresh PDA plate. The cultural characteristics and pathogenicity of mono-ascospore cultures were assessed with the measures described above.

### Transmission of hypovirulence

To test the possible transmission of hypovirulence from strain XG36-1, a non-hypovirulent progeny of strain XG36-1, strain XG36-1A34, was randomly selected for labelling with hygromycin B resistance gene (*hph*) mediated by *Agrobacterium *transformation [29]; and one *hph*-labelled insert which was similar to strain XG36-1A34, named as strain XG36-1A34^R^, was chosen for the transmission test. Then strain XG36-1A34^R ^was dual cultured with strain XG36-1 in a PDA plate allowing the two colonies to intermingle according to Jiang et al [23]. Mycelial agar plugs at the colony margin of strain XG36-1A34^R ^were placed onto fresh PDA containing 50 μg/mL hygromycin (where unlabelled strains could not grow), and placed at 20°C for 3 to 4 days. Mycelial plugs were taken from the new colonies and transferred into fresh PDA plate without any hygromycin. The cultural characteristics and pathogenicity of subcultures of XG36-1A34^R ^after contacting strain XG36-1 were tested with measures described above.

### Transmission electron microscopy (TEM) observation

Strain XG36-1, strain XG-13, strain 36-1A34^R ^and its subcultures after contacting strain XG36-1 were grown on PDA plates for 2–3 days at 20°C, and the mycelia of each strain were collected for TEM observation (FEI Tecnai G^2 ^20 TWIN transmission electron microscope). The approach for TEM observation followed Boland et al [26]. To extract viral particles, strain XG36-1 was grown out on cellophane membranes on top of PDA for 3 days, and mycelia were harvested for extracting virus-like particles (viral particles) according to Ghabrial and Havens [30]. The viral particles were observed under TEM after negative staining with uranyl-acetate. TEM observation was carried out at Institute of Virology, Chinese Academy of Sciences, Wuhan, P R China.

### Extraction and confirmation of dsRNA

Mycelia for dsRNA isolation were grown out on cellophane membranes on top of PDA for 2 to 10 days, respectively. Following harvesting, the mycelium was stored at 80°C before use. The procedure for dsRNA extraction described by Xie et al [14] was used with minor modifications. The RNA sample was first digested with RNase-free DNaseI, treated with S1 nuclease, and then subjected to electrophoretic analysis on 1% agrose gel.

### Data analysis

Each test had three to five replicates, and data from the experiments were analyzed using an analysis of variance (ANOVA) in SAS (SAS Software, NJ). Treatment means were compared with the test of least significant difference (LSD) at the p = 0.05.

## Competing interests

The authors declare that they have no competing interests.

## Authors' contributions

DJ, YF, LZ, JX and GL designed the experimental strategy; LZ and JX conducted the experiments; DJ, LZ, YF, JX, GL and XY were involved in the data analysis and their processing; and DJ and YF wrote the manuscript. All authors approved the final manuscript.
